# An alternative method to amplify RNA without loss of signal conservation for expression analysis with a proteinase DNA microarray in the ArrayTube^® ^format

**DOI:** 10.1186/1471-2164-7-144

**Published:** 2006-06-12

**Authors:** Susann Schüler, Ingrid Wenz, B Wiederanders, P Slickers, R Ehricht

**Affiliations:** 1 Institute of Biochemistry, Klinikum, Friedrich-Schiller-Universität Jena, Germany; 2 Clondiag chip technologies GmbH Jena, Germany

## Abstract

**Background:**

Recent developments in DNA microarray technology led to a variety of open and closed devices and systems including high and low density microarrays for high-throughput screening applications as well as microarrays of lower density for specific diagnostic purposes. Beside predefined microarrays for specific applications manufacturers offer the production of custom-designed microarrays adapted to customers' wishes.

Array based assays demand complex procedures including several steps for sample preparation (RNA extraction, amplification and sample labelling), hybridization and detection, thus leading to a high variability between several approaches and resulting in the necessity of extensive standardization and normalization procedures.

**Results:**

In the present work a custom designed human proteinase DNA microarray of lower density in ArrayTube^® ^format was established. This highly economic open platform only requires standard laboratory equipment and allows the study of the molecular regulation of cell behaviour by proteinases. We established a procedure for sample preparation and hybridization and verified the array based gene expression profile by quantitative real-time PCR (QRT-PCR). Moreover, we compared the results with the well established Affymetrix microarray. By application of standard labelling procedures with e.g. Klenow fragment exo^-^, single primer amplification (SPA) or *In Vitro *Transcription (IVT) we noticed a loss of signal conservation for some genes. To overcome this problem we developed a protocol in accordance with the SPA protocol, in which we included target specific primers designed individually for each spotted oligomer. Here we present a complete array based assay in which only the specific transcripts of interest are amplified in parallel and in a linear manner. The array represents a proof of principle which can be adapted to other species as well.

**Conclusion:**

As the designed protocol for amplifying mRNA starts from as little as 100 ng total RNA, it presents an alternative method for detecting even low expressed genes by microarray experiments in a highly reproducible and sensitive manner. Preservation of signal integrity is demonstrated out by QRT-PCR measurements. The little amounts of total RNA necessary for the analyses make this method applicable for investigations with limited material as in clinical samples from, for example, organ or tumour biopsies. Those are arguments in favour of the high potential of our assay compared to established procedures for amplification within the field of diagnostic expression profiling. Nevertheless, the screening character of microarray data must be mentioned, and independent methods should verify the results.

## Background

Proteinases play an essential role in numerous biological processes such as cell growth and differentiation, embryonic development, wound healing, antigen presentation etc., but also for pathological events like inflammation, coronary heart failure or cancer [1,2]. Above all, tumour invasion and metastatic spread of tumours require the activity of proteolytic enzymes [3]. Some proteinases have been identified as prognostic markers for the overall survival of a patient, as well as an indicator for a metastasis-free outcome post therapy. For instance there is evidence for a positive correlation between the expression of Matrixmetalloproteinases 2 and 9 (Gelatinases A and B) and a poor prognosis in almost all kinds of cancer [4]. Urokinase (uPA), the urokinase receptor and the uPA inhibitors PAI-1 and -2 are enhanced in several female tumours [5-7]. Also, the Cathepsins B, L, K and S may play an active role in cancer development [8] and inflammation diseases like rheumatoid arthritis [9]. Because of the importance of proteolysis *in vivo*, it is of great interest to better understand how the balance between active proteolytic enzymes and their endogenous inhibitors is controlled. Furthermore, it is also important to learn at which level (expression, activation, compartimentalization) a disturbed proteolysis is caused. The introduction of synthetic inhibitors of proteinases as therapy concepts drew high expectations for controlling or limiting the degradative potential of proteinases in e.g. cancer or rheumatoid arthritis [9]. However, several aspects of such treatments are still uncertain. Expression analysis using DNA microarray technology is one potent tool to study the involvement of proteases in various normal and pathological processes or to follow the expression during an inhibitor treatment. However, it must be urgently stressed that such analyses require confirmation by independent methods such as e.g *in vivo *activity measurements of enzymes or at least QRT- PCR measurements.

Recent developments in DNA microarray technology led to a variety of open and closed devices and systems including high and low density microarrays for high-throughput screening applications as well as specific diagnostic purposes [10]. Beside predefined microarrays for specific applications manufacturers offer the production of custom designed microarrays adapted to customers' wishes [11-15].

Array technology demands a complex procedure including both the reproducible and robust array production and the many steps in sample preparation (e.g. RNA extraction, amplification and sample labelling), hybridization and detection which may frequently lead to a high variability of the results between several and even equal experiments. Therefore, standardization and normalization procedures are very important and yet one of the most time-consuming steps in the development of new array based assays.

While analyzing RNA of clinical samples the amount of material available often limits the examination. Direct sample labelling via reverse transcription requires 20–100 μg total RNA [16] making this simple protocol inapplicable for most routine diagnostic processes of clinical samples. Two main concepts offer solutions for this dilemma. One possibility is to increase the labelling efficiency (meaning a higher signal per molecule ratio). This has been achieved using techniques like e.g. tyramide signal amplification and/or amino-allyl labelling [17,18]. Other protocols amplify the RNA necessary for labelling in a different manner. One basic protocol is the amplification of aRNA (antisense RNA) by IVT (*In Vitro *Transcription), the so called Eberwine protocol [19]. A variety of alternatives to this costly and lengthy procedure have been developed: SPA (Single Primer Amplification) [16], SMART technology (Clontech)[20], Ribo-SPIA™ RNA amplification (NuGEN) [21], amplification using terminal continuation (TC RNA amplification) [22] etc. Numerous researchers investigated the conservation of differential expression and reproducibility by comparison of different amplification techniques, standardization and normalization of array results [23-28], respectively.

The main challenge is to maintain the differences of expression levels between different RNA species during the labelling procedure in a reproducible manner. We work with custom designed microarrays of low density in the ArrayTube^® ^format. This offers a cost effective platform and requires only standard laboratory equipment and an array tube reader (atr01). By application of standard labelling procedures (e.g. Klenow fragment exo^-^, single primer amplification or IVT) we noticed a loss of some gene signals. In order to overcome this problem we developed a modified SPA protocol. We included target specific primers designed individually for each spotted target oligomer. Here we present the results by amplifying, labelling and hybridizing specific transcripts of interest.

## Results and discussion

### Comparison of different labelling methods

Labelling in the course of reverse transcription of as much as 5 μg total RNA resulted in non detectable or only very poorly detectable hybridization signals (results not shown). This was expected and corresponds to descriptions in literature and to protocols recommending 25–100 μg total RNA for direct reverse transcription labelling reactions [16]. Therefore, we prepared samples according to the alternative and modified protocols, respectively.

**Figure 1:**
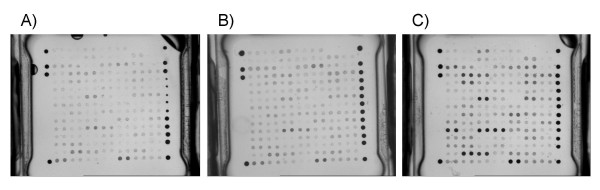
**Labelling und Amplification of RNA from HEK293**. (A) Labelling of initially reverse transcribed c-DNA with Klenow fragment exo^- ^(10 μg total RNA), (B) Amplification of total RNA according the Eberwine protocol [19], Labelling of 2 μg antisense RNA with Klenow fragment exo^-^, (C) Labelling via SPA (0.25 μg total RNA).

Figure 1 shows an example for transcribing and labelling of total RNA extracted from HEK (human embryonic kidney) 293 (ACC # 305) cells according to three different methods: A) Labelling of reverse transcribed cDNA in the course of the reaction with Klenow fragment, B) Labelling with Klenow fragment after amplification of total RNA according to the Eberwine protocol [19] and C) synthesis of double stranded cDNA starting from total RNA and labelling in the course of linear amplification using a so-called SPA-primer. Method A) yields labelled products of sense- and antisense-direction, protocol B) and C) result only in antisense stranded products being complementary to the immobilized oligonucleotides on the array. The overall hybridization patterns generated by the three different methods seem similar but are not identical. The signal intensity was stronger and the differentiation was in the case of method C) more pronounced compared to methods A) and B), despite the relatively small amount of initial total RNA in this experiment. The question was whether the improvement achieved by the SPA method was subject to a better signal sensitivity, to a better signal specificity or to both. In order to further study the differences in the expression profiles, we chose three cell lines with different expression profiles – namely human fibroblasts, DLD-1 and 97TM1 (two established cancer cell lines derived from colon adenocarcinoma, ACC # 278 and non-small cell lung cancer, resp., ACC # 388) – to prepare hybridization samples in two different manners: with Klenow fragment exo^- ^and by preparation of double stranded DNA and labelling in the course of linear amplification using SPA primer both starting from initially reverse transcribed total RNA.

### PCR experiments

**Figure 2:**
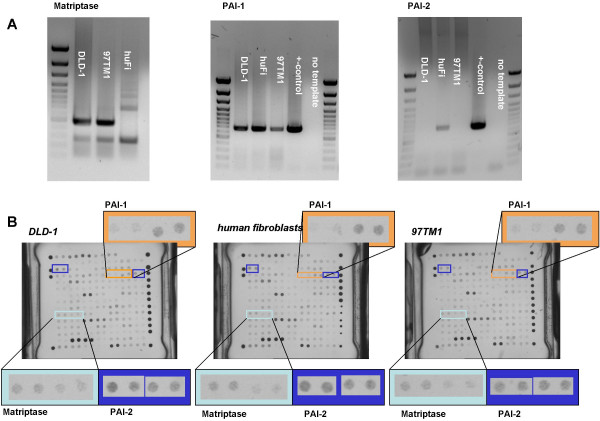
**Comparison of PCR vs. hybridization pattern on the arrays**. A. Electrophoresis of PCR products. Initially reverse transcribed c-DNA of human fibroblasts, DLD-1 and 97TM1 was used as template for PCR with specific primers for Matriptase, PAI-1 and PAI-2. Positive controls: plasmid-cDNAs of the three targets, negative controls were template free. Product sizes: matriptase 470 bp, PAI-1 687 bp, PAI-2 710 bp. B. Array analysis. Sample preparation was performed according to the protocol for overall linear amplification with the SPA primer and simultaneous labelling (see Methods part).

In order to exclude false positive results on the array we reverse transcribed the RNA of the three cell lines into cDNA, which was then used as a template for PCR. A template free control and a positive control were carried along each PCR reaction. A negative result (no signal on the array) should be confirmed by the absence of any PCR product of the expected size (exclusion of contamination by genomic DNA). However, as can be seen in Fig. 2, the PCR results did not completely correspond to the array data. The array showed weak but significant signals for PAI-2 but we did not receive any PCR product for DLD-1 and 97TM1. And the signals for Matriptase were nearly equal in all three cell lines, although human fibroblasts did not show a PCR product. These unexpected results required an explanation. First, we increased the stringency of the washing procedure of the array in order to minimize cross hybridization reactions, but we were unable to change the arrangement and intensity of the hybridization signals due to this. Furthermore, we excluded the possibility of false positive hybridization signals by initially testing all spotted oligos of functionality by hybridization with biotinylated PCR products generated with specific primers from a corresponding plasmid cDNA (results not shown). We did not observe any cross hybridization with the cDNAs generated from plasmids.

Another possibility to explain the results was that they were based by the labelling procedure itself. Therefore, we decided – in contrast to the overall amplification with SPA primer – to try a specific linear amplification as described in the methods section where instead of the SPA primer specific primers for every target were used. Additionally, we incorporated biotinylated dUTP in the linear amplification reaction using the primer mixture and thus, diminished the number of steps required for sample labelling compared to original SPA protocol.

**Figure 3:**
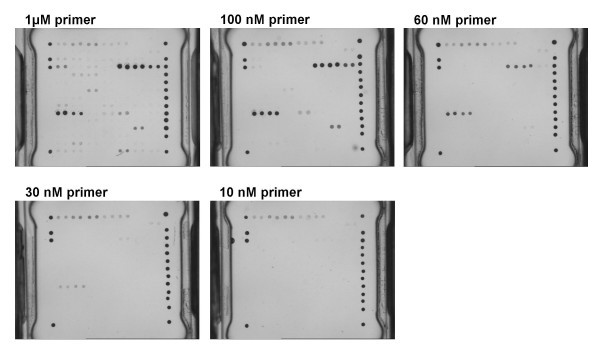
**Dependence of signal development on primer concentration**. ds-cDNA was generated from 0.6 μg total RNA of DLD-1 at 62°C by linear amplification using the primer mix for Matriptase, PAI-1 and PAI-2. Different primer concentrations are indicated. Hybridization to the Array tubes^® ^in accordance to the protocol described in the Methods part.

For orientation, initially six oligonucleotides specific for 3 targets (Matriptase and the PAI-1 and 2) were tested together. These experiments resulted in highly specific hybridization signals matching exactly the expected targets (Fig. 3), however, revealed a strong dependence on the primer concentration.

### Optimization of the labelling protocol with specific primers

We investigated the influence of amplification temperature, primer concentration, reaction volume and ds-cDNA concentration in order to further improve the labelling protocol in the course of linear amplification with specific oligonucleotides. As one example, Fig. 3 shows the signal intensity and hybridization pattern at different primer concentrations at an annealing temperature of 62°C. For further investigations we have chosen a primer concentration of 60 nM each (final). It was applied in all assay investigations to prevent cross reactions and thus, false positive signals. In addition, that primer concentration was the lowest one yielding detectable hybridization signals of all expected signals. The assay was well functioning for that little primer set. This was in accordance with descriptions of a similar procedure to amplify genomic DNA with 39 oligos in a linear manner[14].

### Experiments with an increased number of specific primers

Consequently, the behavior of the system was investigated with a stepwise increasing number of specific primers. Because of the complex reaction mixture cross hybridization reactions may be expected which had to be ruled out.

The experiments were carried out with ds-cDNA from human fibroblasts. In each experiment 100 ng total RNA were used. In a first series of experiments thirty two primers for 16 target genes were used and divided into 4 groups.

-Group 1: Matriptase, PAI-1 and 2 (the initial test mix)

-Group 2: MMP1, 3, 7, 13 and GAPDH

-Group 3: MMP2, 9, 16, β-actin

-Group 4: Cathepsins K and S, TIMP3, TIMP4

The single primer groups were used in amplification reactions. The hybridization patterns were compared with the results of PCR to omit false positive signals. Hybridization signals were confirmed by positive results of the respective PCR measurements. False positive signals were not detected. In the following and final experiment all available specific primers were combined and used for an amplification reaction. We compared the hybridization pattern of the three cell lines human fibroblasts, DLD-1, 97TM1, respectively, already chosen for the first experiments (see above). We noticed a remarkably improved specificity of the hybridization pattern compared to the traditional labelling protocols. The results were in accordance to the results of qualitative PCR experiments – the number of hybridization signals essentially corresponded with the number of positive signals in qualitative PCR.

### Validation of results with real time measurements

**Figure 4:**
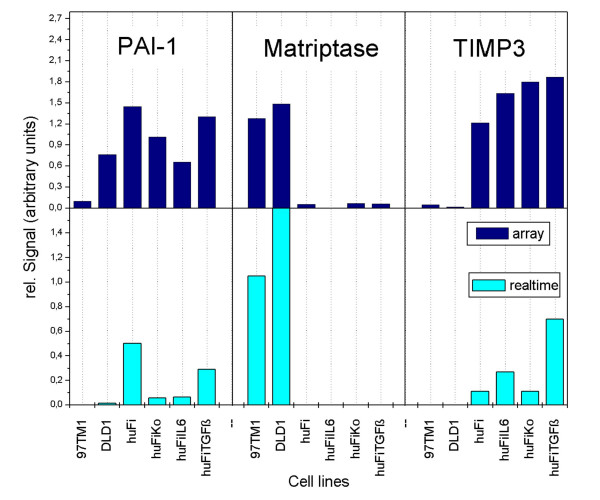
**Comparison of template specific hybridization pattern with QRT- PCR data**. Signal intensities for matriptase, TIMP3 and PAI-1 were normalized to the ß-actin signal. Upper part: array data; lower part: QRT- PCR data. Cell lines: 97TM1, DLD-1, huFi (established line of human fibroblasts), primary synovial human fibroblasts either treated with IL-6 (huFiIL6) and TGFβ (huFiTGFß), respectively, for 24 h in FCS free medium, or non treated (huFiKo). Samples for the array experiments were labelled according to the linear amplification protocol with target specific primers. Amplifications in real time experiments were performed with gene specific primers (primer sequences: matriptase left primer: 5'-gat cct gca aaa ggg tga ga-3', right primer: 5'-cac ttt gga ggc tga gga ag-3'; TIMP3 left primer: 5'-ctt ctg caa ctc cga cat cg-3', right primer 5'-gta gtg ttt gga ctg gta gc-3'; PAI-1 left primer: 5'-gat cga ggt gaa cga gag t-3', right primer: 5'-cac agt gga ctc tga gat g-3').

The expression of selected genes was determined using QRT- PCR in order to confirm the results of the array experiments by an independent method. We have chosen PAI-1, matriptase and TIMP3 because these target genes showed differences in the development of hybridization signals. Figure 4 shows the expression levels of these 3 genes in 6 different samples, normalized to the ß-actin level: 97TM1, DLD-1, an established human fibroblast line, primary synovial fibroblasts treated for 24 h with either TGFβ or IL-6, respectively, or not treated. The normalized signals obtained by QRT- PCR were compared with those of the array experiments. A good overall correspondence was found of the results achieved by the two independent methods.

**Figure 5:**
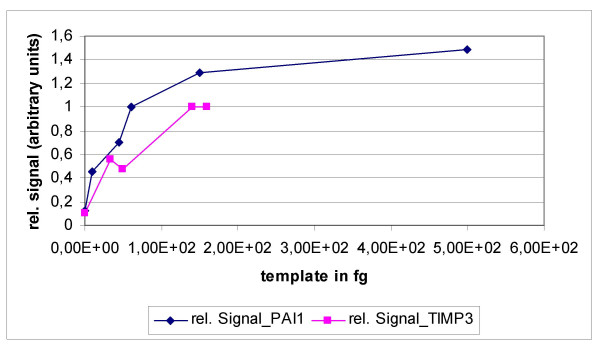
**Relative hybridization signal intensity on the array vs. the absolute gene expression level**. The absolute amounts of TIMP3 cDNA and PAI-1 cDNA were determined by QRT- PCR measurements. These values are presented versus the relative hybridization signals on the array.

**Figure 6:**
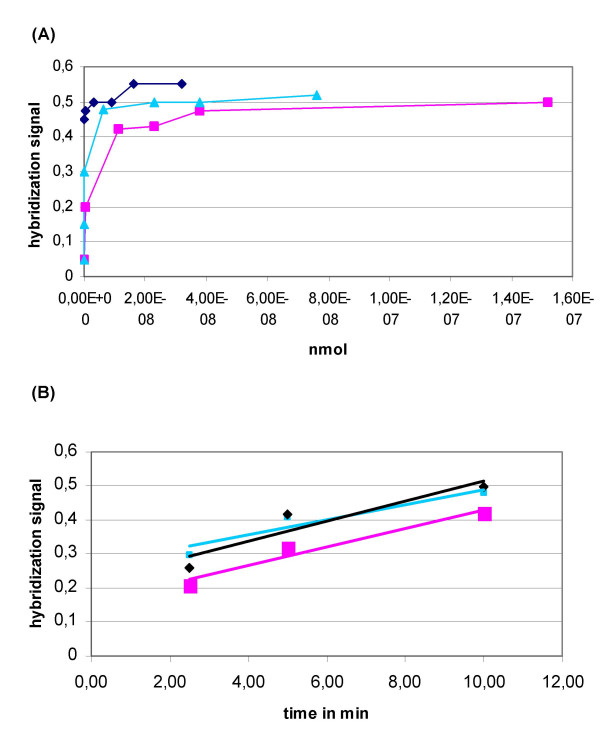
**Hybridization signal intensity on the array obtained with different amounts of synthetic polyA-RNA spiked in**. Synthetic polyA-RNA species were generated using sequences of bacteriophage λ. An oligo (A)_30 _sequence at each 3'end was incorporated in the course of a PCR reaction. Templates for in vitro transcription with T7 RNA polymerase were engineered by cloning in a vector containing a T7 promotor and digesting with HIND III. The transcripts were purified with Oligotex mRNA Minikit (Qiagen, Hilden, Germany) to get pure polyA-transcripts. A) Different amounts of three synthetic polyA-transcripts (B: light blue; H: margenta; J: black) were spiked in as exogenous controls for labelling, hybridization and detection. The hybridization signals determined after 10 min incubation with the HRP substrate are presented versus the amounts of controls spiked in. B) Data analysis at 2.5, 5 and 10 min, respectively, after incubation with the HRP substrate, shown for three different synthetic RNA concentrations (black: 3.2E-9 nmol of sequence J, light blue: 6.48E-9 nmol of sequence B, margenta: 1.14E-8 nmol of sequence H). This way, the dynamic range of array analysis can be expanded to some extent.

However, the graphic points to the qualitative screening character of array experiments, too. We found a good but did not find an absolute conformity between the signals obtained by the different methods. Figure 5 points out one reason for that finding. We calculated a lower limit for the detection on the array of 0.1 fg of PAI-1, Matriptase and TIMP3 cDNA, resp., in the sample (verified by QRT- PCR). We consider this limit also as valid for all other target sequences. The linear range of signal development on the array is limited and was found to be between 1 and 150 fg specific cDNA in the sample. Above this concentration, a further increase in signal intensity can not be reached. The dynamic range of array analyses like this is always limited and does never reach that of QRT-PCR. The spotting of our arrays can only be minimally changed in order to achieve a broader dynamic range due to technical reasons. The detection method (pattern specific precipitation of HRP product and measurement of transmission) is a limiting factor for the dynamic range. However, the enzymatic reaction on the array surface which proceeds in a non linear manner and enhances the hybridization signal allows a very sensitive detection of the signal intensity at every time point during the process of development (see Fig. 6B). This means, the development of rare signals may be seen when high concentrated targets are already in the saturation range. As a consequence of that, the amount of cDNA resulting from the reverse transcription which is used for the specific linear amplification may be enhanced by a factor of 2 or 3 or may be diluted tenfold, respectively, in order to achieve reliable data. We are generally using about 100 ng of cDNA for that purpose, knowing that not all transcription products are complete. Signals of low expressed or incompletely transcribed genes would be lost if only one concentration of cDNA is used. This underlines again the screening character of the array methodology which is further supported by results presented in Fig. 6A. We generated synthetic polyA-RNA from sequences of spike-in controls by in vitro transcription. These different RNA preparations served as control for sample labelling procedures, hybridization and detection. The absolute intensity of hybridization signals was related to the amount of RNA spiked in. Even in this case, we found a region of linear dependence, followed by signal saturation.

Array analyses are extremely dependent on a high reproducibility. Most problems to be solved by an array analysis are based on the direct comparison of a normal sample with samples in which different expression is expected. Therefore, competitive hybridization where two different samples are labelled with e.g. Cy3 and Cy5, respectively, and afterwards mixed and hybridized again one and the same array is the mostly used technique in array based expression profiling. In our experiences the modification of only one part of the assay – e.g. just the polymerase used for amplification – or of the protocol, results in non- or hardly comparable array results. Not only the kind of labelling but also the conditions of the labelling protocol are very decisive for comparable and successful array experiments.

**Figure 7:**
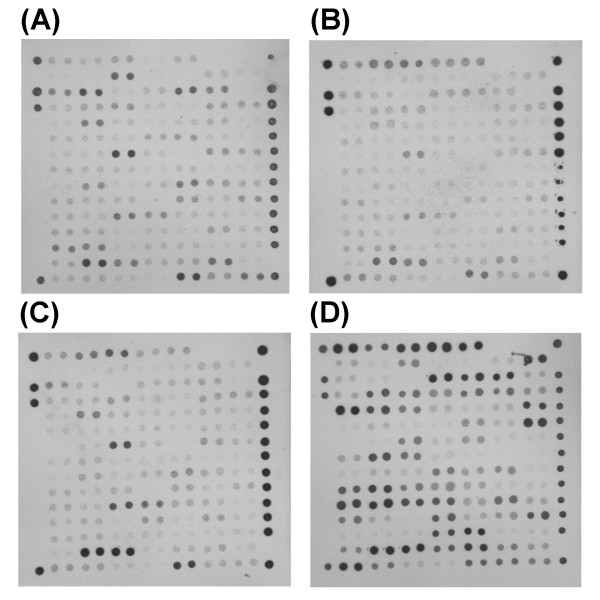
**A direct comparison of the hybridization pattern of samples generated from total RNA of DLD-1 by the different labelling procedures**. (A) Labelling of 20 μg total RNA in the course of reverse transcription (biotinylated Oligo-dT), hybridization for 18 h at 45°C, 1 ng polyA-spike in controls were added. (B) 5 μg total RNA were reverse transcribed and the resulting cDNA was labelled by Klenow fragment exo^-^. (C) Labelling of ds-cDNA generated from 500 ng total RNA in the course of overall linear amplification with the SPA-primer. (D) Labelling of ds-cDNA generated from 100 ng total RNA and 10 pg polyA-spike in controls in the course of linear amplification with the complete set of specific primers according to the protocol described in the Methods part.

The protocol of probe preparation presented in this manuscript is part of a complete evaluated and optimized assay and results in a very specific signal development and a high sensitivity of detection. Figure 7 refer to that fact by direct comparing of the hybridization patterns of samples generated from total RNA of DLD-1 cells by the different labelling procedures. The linear amplification procedure described here in combination with a signal enhancement after hybridization by an enzymatic precipitation reaction at the surface of the array produces a very specific signal beside the remarkably small amount of RNA required. Changes in gene expression of different proteinase RNAs are well detectable, although the validation by independent procedures is absolutely required. The procedure is cost and time effective. The high specifity of signal development does not require an additional RNA purification. Hybridization in array tubes takes not more than 3 hours; it includes a control of signal development by biotinylated markers, and spike-in controls. The in-time registration of signal development makes it possible to analyze the array at a moment which is optimal for any selected target. This option is of importance in cases where some signals are quickly saturated at an early moment of measurement.

### Comparative hybridization experiments – Affymetrix whole genome chip Hu133 plus 2.0 versus tube array

To elucidate the quality of the new protocol we performed comparative hybridization experiments with our assay and with the well-established Affymetrix whole genome chip Hu133. However, only 44 of the 49 targets present on the tube array were also present on the Affymetrix chip. We have chosen a stimulated and a non-stimulated sample (the established colon carcinoma cell line Coga-1 [29] with and without Interleukin-6) to detect differences in the expression profiles and to compare the results achieved using the two different analytic systems. We were aware of the difficulties to compare two absolutely different systems concerning both the probe labelling protocol and the detection method: affymetrix chip results in absolute fluorescence values; array tube system applies a non linear enzymatic precipitation reaction (and thus a signal enhancement) to detect hybridization signals. The consequence is a much higher dynamic range for the affymetrix chip than the array tube may achieve. Nevertheless, the gene expression profile should be the same applying the different array systems, subject to the condition of high quality of analyses.

**Figure 8:**
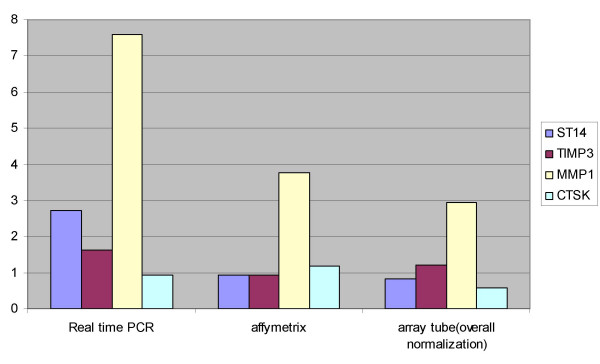
**Change of the expression levels of 4 genes in Coga cells after stimulation with IL-6 analysed by three different methods**. Four expressed genes as determined on the Affymetrix chip, the array tube and by quantitative RT-PCR were considered. The ratio values (non stimulated vs. IL-6 stimulated Coga cells) are indicated. (The ratio Coga/Coga + IL-6 results in high values for down regulated genes after treatment with IL-6).

Only one gene (MMP-1) was significantly down regulated and that was found with both systems and was additionally confirmed by quantitative RT-PCR (Figure 8).

With one exception, all expressed genes detected with the Affymetrix chip (cut off: fluorescence value of 300, detection p-value < 0.065) were also found with the array tube, only MMP-14 which showed very high fluorescence values on the Affymetrix chip was not detectable at the array tube. However, the quantitative RT-PCR measurement confirmed the result of the array tube (no expression of MMP-14).

On the other hand, some hybridization signals which were present at the array tubes were absent on the Affymetrix system (32 hits on the tube array vs. 21 on the Affymetrix system). In order to explain the differences we checked five of the positive array hits with PCR and quantitative RT-PCR, respectively. Table 1 summarizes the results for the non-stimulated cell line. The expression levels of all genes listed in Table 1 were close to the detection limit of quantitative RT-PCR or were detected as only faint bands at the agarose gel, respectively.

Furthermore, we tried to correlate the hybridization signal strength of genes with the absolute amount of RNA determined by quantitative RT-PCR. This result is illustrated in Table 2 for five genes. The expression level of ST14 is one order of magnitudes higher than that of MMP-1 as determined by RT-PCR, however, the relative fluorescence values obtained using the Affymetrix chip gave a 6-fold higher value for MMP-1. A similar phenomenon was also found using the array tube: whereas PAI-I was found to be 20 times less expressed as Cathepsin K, the tube array indicated only a 2-fold higher expression of Cathepsin K than of PAI-I. These findings again point to the screening character of array experiments.

However, the main application of array analyses is the direct comparison of two samples as stimulated vs. non-stimulated cells or normal vs. tumor tissue etc. For this purpose, we found a good correlation of the expression profiles determined with the two microarray systems/probe preparation protocols, respectively, and the quantitative RT-PCR results.

## Conclusion

We designed a protocol for amplifying mRNA in a linear mode from as little as 100 ng total RNA. The protocol offers a simple method for detecting even low expressed genes by microarray experiments in a reproducible and sensitive manner. The central point of the method is the linear ssDNA amplification step with concomitant labelling. Preservation of signal integrity and the little amounts of total RNA necessary for this labelling protocol make that method applicable for investigations with limited amounts of material like clinical samples from biopsies or tumours.

## Methods

### DNA microarray design and preparation

For the manufacturing of the arrays, 3'-aminomodified oligonucleotides were purchased from Metabion (Martinsried, Germany). Oligonucleotides were used at a final concentration of 10 μM in Spotting Buffer 1 (CLONDIAG chip technologies GmbH, Jena, Germany) and spotted using a Microgrid II spotting machine (Genomic Solutions Ltd., UK) following the procedure supplied by the manufacturer. Every probe was spotted redundantly two times on the array. After production, arrays were inserted into ArrayTube™ reaction vials.

Probe sequences were derived from published sequences using the Array Design software package by Clondiag Chip Technologies (Jena, Germany). In the additional files 1, 2, 3 tables show target genes, sources of the sequence data, as well as probe sequences and array layout. Consensus regions in the alignments of all available sequences of each target were chosen for the probe design. The resulting sequences were selected to be specific for the target and to have similar length, GC content and melting temperatures in order to yield comparable signal intensities. The final probe sequences were again blasted against the database [30] to exclude false-positive reactions due to possible cross-reactivities or false-negative reactions due to sequence variations.

The site directed oligonucleotide set for the linear amplification procedure consisted of 94 oligonucleotides. It was designed according to the initial alignments for the probe design as described above. For each target, a consensus region was identified which was situated 5 to 50 bp upstream of the probe binding site (see additional files). Sequences with similar physicochemical parameters were chosen from these regions and used for primer design. The final primer sequences were blasted against the database [30] avoiding possible cross-reactivities as well as sequence variations. Primer sequences are also listed in additional files (additional file 4). Oligonucleotides were purchased from Invitrogen (UK). They were used as a stock solution mixture with concentrations of 1 μM for every individual primer.

The development of the Affymetrix microarray was performed in an independent routine lab according to the manufacturers recommendations. 5 μg purified RNA was used as starting material for this purpose.

### RNA preparation

Total RNA from cells and tissues was isolated with TRIZOL^® ^Reagent (Invitrogen, UK) according to the suppliers' instruction. RNA was re-dissolved in RNase-free water. The quality of the isolated RNA was controlled using non denaturing 1.25% agarose gel electrophoresis and the determination of the A_260_/A_280 _ratio (GeneQuant II, Pharmacia Biotech). Only samples exhibiting no RNA degradation and showing an A_260_/A_280 _ratio equal or above 1.8 were used for further applications. Preparations were stored at -80°C.

### Labelling by reverse transcription of total RNA

For direct labelling of cDNA via reverse transcription 10 μg of total RNA was mixed with 1.5 μl oligo-dT^15^-primer (0.5 μg/μl, Promega, Mannheim, Germany) and sterile water (final volume 14, 6 μl), denatured for 15 minutes at 65°C, then chilled on ice for 5 minutes. After a short centrifugation step a mastermix consisting of 6 μl 5× First Strand Buffer (Invitrogen), 2 μl 0.1 M DTT (Invitrogen), 0.7 μl RNaseOUT (Invitrogen, 40 U/μl), 2.5 μl 5 mM dNTPs (dATP, dCTP, dGTP, Fermentas), 1 μl 3.25 mM dTTP, 1.4 μl 1 mM Biotin-16-dUTP (ROCHE, Penzberg, Germany) and 1.8 μl MMLV-RT (200 U/μl, Invitrogen) was added to get a final volume of 30 μl. The reaction mixture was incubated for 2 h at 37°C, and afterwards stopped by adding of 1 μl 200 mM EDTA (final concentration ca. 7 mM). The sample was precipitated with 0.1 vol 4 M LiCl/2.5–3 vol ice cold absolute ethanol, washed with ice cold 70% ethanol and resuspended in 10 mM EDTA.

### Labelling with Klenow fragment

After First Strand cDNA synthesis with oligo-dT^15^-primer (Promega) and MMLV-RT (Invitrogen) the resulting cDNA was precipitated with LiCl/ice cold absolute ethanol, washed with ice cold 70% ethanol and dried. The pellet was resuspended in 68 μl deionisized water. This mixture served as template for a labelling reaction with Klenow-Fragment, exo^-^. We used the BioLabel DNA-Labelling Kit (Fermentas) and followed the protocol provided by the manufacturer to generate a biotin labeled hybridization sample. This DNA was purified by precipitation (LiCl/Ethanol) for further applications.

### Eberwine protocol

The First Strand cDNA was prepared with an oligo-dT T7-primer (5'-AAA CGA CGG CCA GTG AAT TGT AAT ACG ACT CAC TAT AGG CGC TTT TTT TTT TTT TTT TTT TTT TTT-3') and Superscript II RT (200 U/μl, Invitrogen) in a thermal cycler (10 minutes at 20°C, 60°C minutes at 37°C, final volume 10 μl). To generate the second strand an ice cold mastermix of 52.75 μl water, 7.5 μl 10×Second Strand buffer, 1.5 μl 10 mM dNTPs, 1 μl E. coli DNA-Ligase (5 U/μl, Fermentas), 2 μl DNA polymerase I (10 U/μl, Fermentas) and 0.25 μl RNase H (4.5 U/μl, Fermentas) was added to the cooled first strand tubes (final volume 75 μl). The double stranded cDNA (ds-cDNA) was purified by extraction with one volume phenol (once) and one volume of a chloroform/isoamyl alcohol mixture (24:1, once) and precipitated with 8 M NH_4_-acetate/ethanol at room temperature, washed twice with ice cold 70% ethanol and dried. The pellet was resuspended in 4 μl water. For In Vitro Transcription we used the Ambion Megascript kit (Ambion, USA). All components except the enzyme were allowed to come to room temperature. 1 μl of each component was added to the 4 μl ds-cDNA preparation and incubated for 16 h at 37°C. The resulting antisense RNA was purified with the RNeasy mini kit (Qiagen, Hilden, Germany) according to the manufacturer's protocol.

### SPA

Single primer amplification (SPA) and labelling with Klenow fragment exo^- ^was performed according to the original protocol published in [16].

### Labelling in the course of linear amplification reactions

Double stranded cDNA generated according to the Eberwine protocol described above served as template for labelling during the linear amplification reactions. For overall amplification we used a primer with the sequence of the T7-promoter (called SPA primer, analogue to the SPA-protocol).

For specific amplification of selected targets we used amounts of ds-cDNA-preparations representing original amounts of 50–100 ng total RNA. An ice cold mixture of water, 1.5 μl MgCl_2 _(50 mM), 5 μl 10×PCR buffer (Invitrogen), 1.5 μl 5 mM dNTPs (dATP, dCTP, dGTP), 0.5 μl 3.25 mM dTTP, 0.86 μl 1 mM Biotin-16-dUTP (ROCHE), 0.6 μl Taq polymerase (5 U/μl, Invitrogen) and 3 μl primer mix (1 μM each) resulting in a final primer concentration of 60 nM each was added to the template (final volume 50 μl). The amplification was performed in a thermal cycler according to the following protocol: 3 min 94°C, 40 cycles: 1 min 94°C, 1 min 62°C, 2 min 72°C, cooling to 4°C. The reaction mixture was divided to get material for duplicates and used for hybridization without further purification steps.

### Hybridization and detection

The array tube was conditioned by washing three times with 500 μl distilled water and once with 500 μl hybridization buffer 1 (Clondiag, Jena) at 25°C for 5 minutes. All steps were carried out using a horizontal shaker with temperature regulation (550 rpm, Thermomixer compact, Eppendorf, Germany). Biotinylated spike controls (0.05 μl each, equivalent to 6.7 × 10^-6^-1 × 10^-5 ^nmol) were added to the samples as external control for hybridization, conjugation of enzyme and signal development due to TMB precipitation.

The samples were denatured at 95°C for 10 min and briefly centrifugated at 13000 rpm to collect the sample. 200 μl of pre-warmed hybridization buffer (50°C) was added to each sample and the resulting mixture transferred into the array tube. Hybridization was allowed to proceed on the shaker for 3 h at 50°C and 550 rpm.

The array tube was washed with 500 μl 2×SSC/0.01% Triton (5 min, 30°C), 2×SSC (5 min, 20°C) and 0.2×SSC (5 min, 20°C). After blocking with 100 μl of a blocking solution (2% milk powder, freshly prepared in 6×SSPE/0.005% Triton, 15 min, 30°C) the array was incubated for 15 min at 30°C with 100 μl of a 1:10 000 dilution of HRP Streptavidin (1 mg/ml, Clondiag, Jena) freshly prepared in 6×SSPE/0.005% Triton. Following that the array was washed twice with 500 μl 2×SSC/0.01 Triton (2 min, 30°C), twice with 500 μl 2×SSC and once with 0.2×SSC (5 min, 20°C).

The peroxidase pecipitation reaction (100 μl peroxidase substrate, Clondiag, Jena) was monitored by the ATR01 array tube reader (Clondiag, Jena) at 25°C recording 60 images (one image per 10 sec). Data analysis was carried out using IconoClust software Version 2.2 (Clondiag) determining the signal intensity and the local background value of each spot. The local background absorbance was subtracted from the absorbance of the spots. Only the average values of redundant spot hybridization signals with amounts above 0.05 (mean – local background values) were considered as positive. Both spotted oligos of an examined gene had to be "positive" to be considered as expressed. If these conditions were met, the signal resulting from the hybridization with the oligo sequence situated closer at the 3'end of the RNA sequence was used for further calculations.

### Real time PCR (QRT-PCR) experiments

QRT- PCR experiments were carried out using a MyiQ™ Single colour QRT- PCR detection system (BIORAD, Herculas, CA). Reaction mixtures contained: 1 μl cDNA, 9.5 μl water, 2 μl primer mix (sense and antisense, 10 μM each), and 12.5 μl iQ SYBR Green Supermix (BIORAD, final volume 25 μl). A dilution series (10^-2 ^- 10^-6^ng) of the specific PCR product of interest was prepared to determine the standard curve (absolute quantification). Template free controls served as a test of primer quality (formation of dimers etc.). First of all the melting curve of each target was measured to determine the optimal temperature for real time analysis (e.g. ß-actin: 72°C, PAI-1: 83°C, matriptase: 89°C, TIMP3: 85°C). The samples were amplified according to the following protocol: 3 min 95°C, 35 cycles: 20 sec 95°C, 40 sec 58°C, 1 min 72°C, real time data registration for 8 sec at the specific temperature determined before for each target. All samples were measured in duplicates and the right formation of the products was verified by agarose gel electrophoresis (1% agarose, unknowns, standards and no template control, product sizes: matriptase 470 bp, PAI-1 687 bp, TIMP3 445 bp). Real time data analysis was carried out using the optical system software version 1.0 supplied with the MyiQ™ real time instrument (BIORAD).

### Stimulation experiments

Primary human fibroblasts were isolated from arthritic patients according to the rules of the ethic commission of the Friedrich Schiller University Jena. Cells were cultured in DMEM/high glucose, 10% FCS, gentamycine 0.5 ml/100 ml (in triplicate, 75 cm^2 ^flasks) until 80% confluence was reached. The medium was removed; the cells were washed twice with FCS free medium and further cultured in FCS free medium overnight. The medium was then removed, and 4 ml of fresh FCS free medium was used for stimulation experiments. The first sample served as control (FCS free medium without any cytokine. The second sample was stimulated with 20 ng IL6/ml medium, the third sample was stimulated with 2 ng TGFß/ml medium. Stimulation was performed for 24 hours. Medium was removed, cells were harvested and RNA was isolated with TRIZOL^® ^reagent as described above. RNA quality was checked by non denaturing agarose gel electrophoresis and used for preparation of double stranded cDNA according to the first steps of the Eberwine protocol (see above). Additionally, an established human fibroblast line was also used for expression analysis.

## Abbreviations

aRNA antisense RNA

DMEM Dulbeccos Minimum Essential Medium

ds-cDNA double stranded cDNA

DTT Dithiothreitol

EDTA ethylenediaminetetraacetic acid

FCS Fetal Calf Serum

HRP Horse Radish Peroxidase

IVT In Vitro Transcription

MMLV-RT Moloney Murine Leukemia Virus Reverse Transcriptase

PCR Polymerase Chain Reaction

QRT-PCR Quantitative Real Time PCR

rpm rounds per minute

SPA Single Primer Amplification

SSC Sodium Saline Citrate

SSPE Sodium Saline Phosphate Ethylenediaminetetraacetic acid

TMB 3, 3', 5, 5'-Tetramethylbenzidin

## Authors' contributions

SS and IW carried out most of the practical work at the bench. SS drafted the manuscript.

PS calculated the optimal primer and target sequences.

RE developed the tube array technology at Clondiag and helped to draft the manuscript.

BW was the supervisor of the project and participated in the planning and design of the experiments. He helped to draft the manuscript.

All authors read and approved the final manuscript.

## Supplementary Material

Additional file 1 List of target sequences Click here for file

Additional file 2 Layout array Click here for file

Additional file 3 Primer sequences Click here for file

Additional file 4 Probes with reference sequences Click here for file

## Figures and Tables

**Table 1:** Hybridization signal strength of five genes found to be expressed using the array tube but not using the Affymetrix chip, respectively, compared with the results of quantitative PCR experiments (non-stimulated Coga cells). Array tube: relative transmission values, normalized overall. Affymetrix chip: relative fluorescence value, normalized overall

Gene	Affymetrix chip	Array tube	PCR	Quantitative RT-PCR
PAI-1	not present	0,53		4,34*10^-7 ^ng
MMP7	not present	0,52		1,02*10^-6 ^ng
MMP8	not present	0,47	positive	
CST5	not present	0,89	positive	
CTSK	273,8^+^)	0,43		8,13*10^-6 ^ng

^+^) this value is below the cut off of 300.

**Table 2:** Relative expression level of selected genes (fluorescence and transmission values, respectively) versus expression level determined by quantitative RT-PCR (non stimulated cell line)

gene	Affymetrix chip	Array tube	Quantitative RT-PCR
ST14	1000; 3000	1,6	6*10^-3 ^ng
MMP1	6320,6	1,2	3,6*10^-4 ^ng
TIMP3	619; 976; 1564,5	1,15	2,2*10^-4 ^ng
CTSK	273,8 ^+^)	0,43	8,13*10^-6 ^ng
PAI-1	Not present	0,84	4,34*10^-7 ^ng

^+^) this value is below the cut off of 300.
