# Supplementary material for: An alternative method to amplify RNA without loss of signal conservation for expression analysis with a proteinase DNA microarray in the ArrayTube® format
Source: BMC Genomics. 2006 Jun 12;7:144. doi: 10.1186/1471-2164-7-144 (PMC1526438; doi:10.1186/1471-2164-7-144)
Supplement: Additional file 1 — List of target sequences [file 1471-2164-7-144-S1.pdf]

Additional file 1 – table 1

List of target sequences

i	locusID	geneSymbol	description	refSeq_mRNA	CDS_start	CDS_end	cDNA_start	cDNA_end
1	60	ACTB	actin_beta	NM_001101.2	74	1201	677	1104
2	2152	F3	tissue_factor	NM_001993.2	124	1011	218	616
3	2597	GAPD	GAPDH	NM_002046.2	76	1083	678	947
4	1508	CTSB	Cathepsin_B	NM_001908.2	152	1171	127	609
5	1075	CTSC	Cathepsin_C	NM_001814.2	99	1490		
6	1510	CTSE	Cathepsin_E	NM_001910.2	119	1309		
7	1509	CTSD	Cathepsin_D	NM_001909.3	134	1273	424	1318
8	8722	CTSF	Cathepsin_F	NM_003793.2	91	1545	305	886
9	1511	CTSG	Cathepsin_G	NM_001911.2	38	805		
10	1512	CTSH	Cathepsin_H	NM_004390.2	96	1103	452	1097
11	1513	CTSK	Cathepsin_K	NM_000396.2	125	1114	529	1042
12	1514	CTSL	Cathepsin_L	NM_001912.2	345	1346	254	865
13	1520	CTSS	Cathepsin_S	NM_004079.3	134	1129	237	900
14	1522	CTSZ	Cathepsin_Z	NM_001336.2	126	1037	463	983
15	6768	ST14	Matriptase	NM_021978.2	153	2720		
16	4312	MMP1	MMP_1	NM_002421.2	72	1481	532	1208
17	4313	MMP2	MMP_2	NM_004530.1	290	2272	1917	2566
18	4314	MMP3	MMP_3	NM_002422.2	64	1497	551	1177
19	4316	MMP7	MMP_7	NM_002423.2	48	851	123	641
20	4317	MMP8	MMP_8	NM_002424.1	72	1475		
21	4318	MMP9	MMP_9	NM_004994.1	20	2143	1521	2130
22	4319	MMP10	MMP_10	NM_002425.1	23	1453		
23	4320	MMP11	MMP_11	NM_005940.2	23	1489	1101	1689
24	4321	MMP12	MMP_12	NM_002426.1	13	1425		
25	4322	MMP13	MMP_13	NM_002427.2	29	1444	1232	2052
26	4323	MMP14	MMP_14	NM_004995.2	235	1983	438	887
27	4324	MMP15	MMP_15	NM_002428.1	49	2058	327	795
28	4325	MMP16	MMP_16	NM_005941.2	113	1936	82	479
29	4326	MMP17	MMP_17	NM_016155.2	100	1920	338	908
30	4327	MMP19	MMP_19	NM_002429.2	109	1635	1064	1792
31	10893	MMP24	MMP_24	NM_006690.2	4	1941	612	1269
32	7076	TIMP1	TIMP_1	NM_003254.1	63	686	223	723
33	7077	TIMP2	TIMP_2	NM_003255.2	303	965	506	1037
34	7078	TIMP3	TIMP_3	NM_000362.3	1189	1824	1290	1734
35	7079	TIMP4	TIMP_4	NM_003256.1	60	734	434	968
36	5328	PLAU	uPA	NM_002658.1	77	1372	149	794
37	5329	PLAUR	uPAR	NM_002659.1	427	1434	501	1408
38	5054	SERPINE1	PAI1	NM_000602.1	76	1284	1119	1805
39	5055	SERPINB2	PAI2	NM_002575.1	73	1320	341	1052
40	1475	CSTA	Cystatin_A	NM_005213.2	58	354	27	414
41	1476	CSTB	Cystatin_B	NM_000100.2	110	406	175	554
42	1471	CSTC	Cystatin_C	NM_000099.2	76	516	265	641
43	1473	CST5	Cystatin_D	NM_001900.2	5	433		
44	3827	KNG	Kininogen	NM_000893.1	50	1333		
45	-	spikeB	spikeB	spikeB.fna	1	1253		
46	-	spikeH	spikeH	spikeH.fna	1	731		
47	-	spikeJ	spikeJ	spikeJ.fna	1	1750		
