# Supplementary material for: An alternative method to amplify RNA without loss of signal conservation for expression analysis with a proteinase DNA microarray in the ArrayTube® format
Source: BMC Genomics. 2006 Jun 12;7:144. doi: 10.1186/1471-2164-7-144 (PMC1526438; doi:10.1186/1471-2164-7-144)
Supplement: Additional file 2 — Layout array [file 1471-2164-7-144-S2.pdf]

Additional file 2 – table 2 Layout array

* 104<br>(1)	99 spike B	99 spike B	100 spike H	100 spike H	101 spike H	101 spike H	102 spike J	102 spike J	103 spike J	103 spike J					*104
(2)	92 CystC	92 CystC	93 CystD	93 CystD	94 CystD	94 CystD	95 Kng	95 Kng	96 Kng	96 Kng	97 Kng	97 Kng	98 spike B	98 spike B	
* 104<br>(3)	85 PAI 2	85 PAI 2	86 CystA	86 CystA	87 CystA	87 CystA	88 CystB	88 CystB	89 CystB	89 CystB	90 CystC	90 CystC	91 CystC	91 CystC	*104
*104<br>(4)	78 uPA	78 uPA	79 uPA	79 uPA	80 uPAR	80 uPAR	81 uPAR	81 uPAR	82 PAI 1	82 PAI 1	83 PAI 1	83 PAI 1	84 PAI 2	84 PAI 2	*104
(5)	71 TIMP 1	71 TIMP 1	72 TIMP 2	72 TIMP 2	73 TIMP 2	73 TIMP 2	74 TIMP 3	74 TIMP 3	75 TIMP 3	75 TIMP 3	76 TIMP 4	76 TIMP 4	77 TIMP 4	77 TIMP 4	*104
(6)	64 MMP 17	64 MMP 17	65 MMP 17	65 MMP 17	66 MMP 19	66 MMP 19	67 MMP 19	67 MMP 19	68 MMP 24	68 MMP 24	69 MMP 24	69 MMP 24	70 TIMP 1	70 TIMP 1	*104
(7)	57 MMP 13	57 MMP 13	58 MMP 14	58 MMP 14	59 MMP 14	59 MMP 14	60 MMP 15	60 MMP 15	61 MMP 15	61 MMP 15	62 MMP 16	62 MMP 16	63 MMP 16	63 MMP 16	*104
(8)	50 MMP 10	50 MMP 10	51 MMP 10	51 MMP 10	52 MMP 11	52 MMP 11	53 MMP 11	53 MMP 11	54 MMP 12	54 MMP 12	55 MMP 12	55 MMP 12	56 MMP 13	56 MMP 13	*104
(9)	43 MMP 3	43 MMP 3	44 MMP 7	44 MMP 7	45 MMP 7	45 MMP 7	46 MMP 8	46 MMP 8	47 MMP 8	47 MMP 8	48 MMP 9	48 MMP 9	49 MMP 9	49 MMP 9	*104
(10)	36 Matr	36 Matr	37 Matr	37 Matr	38 MMP 1	38 MMP 1	39 MMP 1	39 MMP 1	40 MMP 2	40 MMP 2	41 MMP 2	41 MMP 2	42 MMP 3	42 MMP 3	*104
(11)	29 CathL	29 CathL	30 CathL	30 CathL	31 CathS	31 CathS	32 CathS	32 CathS	33 CathS	33 CathS	34 CathZ	34 CathZ	35 CathZ	35 CathZ	*104
(12)	22 CathF	22 CathF	23 CathG	23 CathG	24 CathG	24 CathG	25 CathH	25 CathH	26 CathH	26 CathH	27 CathK	27 CathK	28 CathK	28 CathK	*104
(13)	15 CathE	15 CathE	16 CathE	16 CathE	17 CathE	17 CathE	18 CathD	18 CathD	19 CathD	19 CathD	20 CathF	20 CathF	21 CathF	21 CathF	*104
(14)	8 Tiss Fact	8 Tiss Fact	9 GAP DH	9 GAP DH	10 GAP DH	10 GAP DH	11 CathB	11 CathB	12 CathB	12 CathB	13 CathC	13 CathC	14 CathC	14 CathC	
*104<br>(15)	1 ßAct	1 ßAct	2 ßAct	2 ßAct	3 Tiss Fact	3 Tiss Fact	4 Tiss Fact	4 Tiss Fact	5 Tiss Fact	5 Tiss Fact	6 Tiss Fact	6 Tiss Fact	7 Tiss Fact	7 Tiss Fact	*104
