# Supplementary material for: An alternative method to amplify RNA without loss of signal conservation for expression analysis with a proteinase DNA microarray in the ArrayTube® format
Source: BMC Genomics. 2006 Jun 12;7:144. doi: 10.1186/1471-2164-7-144 (PMC1526438; doi:10.1186/1471-2164-7-144)
Supplement: Additional file 3 — Primer sequences [file 1471-2164-7-144-S3.pdf]

Additional file 3 – table 3

Primer sequences

No	name	sequence	length	GC	Tm
1	ACTB:942:20	GTTGGCGTACAGGTCTTTGC	20	55	57.1
2	ACTB:1181:21	CTAGAAGCATTTGCGGTGGAC	21	52	56.9
3	CST5:261:22	GTTCGACCGAACTTCACATTGA	22	45	56.5
4	CST5:367:22	CCAGGGAACTTCATTGATCTGG	22	50	56.0
5	CSTA:162:22	CTGCACAGCTTCCAATTTTCCA	22	45	57.3
6	CSTA:326:20	CGTCAGCTCGTCATCCTTGT	20	55	57.5
7	CSTB:258:24	GCACCTTGATGAAGTAGTTTGTCC	24	46	57.4
8	CSTB:367:21	TCATGCTTGGCTTTGTTGGTC	21	48	57.0
9	CSTC:361:20	GTCTTGGTACACGTGGTTCG	20	55	56.3
10	CSTC:485:21	GACAGGTGGATTTCGACAAGG	21	52	56.3
11	CTSB:283:21	TCCACGTTGTAGAAGTTGTGC	21	48	56.1
12	CTSB:1147:23	AGATCTTTTCCCAGTACTGATCG	23	43	55.0
13	CTSC:1112:24	TGGTACTCAGAGGAGTAATAACGA	24	42	55.5
14	CTSC:1468:24	CCTACAATTTAGGAATTGGTGTGG	24	42	55.1
15	CTSD:980:19	CCTCCTTGCACAGGGTCAG	19	63	57.3
16	CTSD:1239:20	GATGTCCATGCCCATGAAGC	20	55	56.7
17	CTSE:432:21	GGCTAGTGCAGTACACAGAGG	21	57	57.5
18	CTSE:1326:18	GTCAGACAGGCAGGCACA	18	61	56.9
19	CTSF:824:24	GGAGAGTATTCAGGTAGATAGTGC	24	46	55.0
20	CTSF:1503:19	CATGGTGTTCACGCCACAG	19	58	56.8
21	CTSG:425:19	GTCCCTCCTGGGCTCTAGG	19	68	57.7
22	CTSG:776:21	GGTCTCCATCTGATCCAGCAG	21	57	57.2
23	CTSH:757:21	CATCCTTGACAAAGCCGATGG	21	52	56.9
24	CTSH:1087:19	GCTCACACCAGAGGGATGG	19	63	57.1
25	CTSK:908:24	GCAGCTTTCATCATAATACACACC	24	42	55.8
26	CTSK:1019:23	CCTTTGTTTCCCCAGTTTTCTCC	23	48	57.2
27	CTSL:762:22	GAGCACCAGTAGCACTAAAAGC	22	50	56.9
28	CTSL:1330:19	GCTCACACAGTGGGGTAGC	19	63	57.7
29	CTSS:584:21	TCCTGTTTTCAGCTTCAGCTG	21	48	56.1
30	CTSS:1082:24	GCTAGCAATCCCACAATGATTTCC	24	46	57.8
31	CTSZ:817:20	TGTGGTGTCCTGGTATTCGG	20	55	56.7
32	CTSZ:1040:20	CTGCGCTTCTAGTGACATGG	20	55	56.1
33	F3:567:22	CACTTTTGTTCCCACCTGTTCA	22	45	56.5
34	F3:1002:24	CAACAGTGCTTCCTTTATGAAACA	24	38	55.5
35	GAPD:899:19	ACCTGGTGCTCAGTGTAGC	19	58	56.6
36	GAPD:1063:19	ACTCCTTGGAGGCCATGTG	19	58	56.6
37	KNG:1048:22	ACAGCTTTCGGTCAACTCTTCA	22	45	57.4
38	KNG:1208:23	CCTATTCGTGATGATCGGAAAGG	23	48	56.3
39	MMP10:790:21	CCGTAGAGAGACTGAATGCCA	21	52	56.5
40	MMP10:1435:21	GCCTAGCAATGTAACCAGCTG	21	52	56.7
41	MMP11:1271:19	GCACGGGACTGTCTACACG	19	63	57.8
42	MMP11:1442:21	ACAGCCAAAGAAGTCAGGACC	21	52	57.5
43	MMP12:920:21	CCTTCAGCCAGAAGAACCTGT	21	52	57.2
44	MMP12:1359:24	CGTTGGAGTAGGAAGTCATATTCA	24	42	55.4
45	MMP13:831:21	GGTTGGGGTCTTCATCTCCTG	21	57	57.3
46	MMP13:1472:20	ACCCCAAATGCTCTTCAGGA	20	50	56.2
47	MMP14:642:20	CCTCGTATGTGGCATACTCG	20	55	55.3

48	MMP14:1902:23	GTCTGAAGAAGAAGACTGCAAGG	23	48	56.6
49	MMP15:504:20	GGTACCAGCCCAACTTCTCC	20	60	57.3
50	MMP15:2070:19	CTGAGCACCGTTAGCAGGA	19	58	56.7
51	MMP16:422:21	GCTACCTCTTGTCTGGTCAGG	21	57	57.1
52	MMP16:1920:20	ACATCACACCCACTCTTGCA	20	50	56.8
53	MMP17:768:21	TCATCGTCAAAGTGGGTGTCC	21	52	57.6
54	MMP17:1662:18	CCACACACCAGCCAGTCC	18	67	57.6
55	MMP19:1318:19	CAGTTCGGGCTAGCTCGTC	19	63	57.6
56	MMP19:1622:23	GGTGAGCAGTCAGTATTCAAACG	23	48	57.2
57	MMP1:1177:21	GCTTCACAGTTCTAGGGAAGC	21	52	56.0
58	MMP1:1349:24	CCATCTTTCATGAAAACTGCATCA	24	38	55.6
59	MMP24:1213:19	CTCGGTTATTGCGCAGACG	19	58	56.8
60	MMP24:1905:21	ACTGGCCGCTTATAGTAGGTG	21	52	56.6
61	MMP2:2054:24	CTTCTTCACCTCATTGTATCTCCA	24	42	55.3
62	MMP2:2295:20	GAAGGCAGTGGAGAGGAAGG	20	60	57.0
63	MMP3:784:22	GAGTCAGGTCTGTGAGTGAGTG	22	55	57.3
64	MMP3:1414:22	ACTCCAACTGTGAAGATCCAGT	22	45	56.3
65	MMP7:341:22	AGTCCATTTTGGGCTATTTGGA	22	41	55.2
66	MMP7:735:23	CCATAGGTTGGATACATCACTGC	23	48	56.2
67	MMP8:805:22	TCTTGAGGGAGTGAGTAGTTGC	22	50	56.7
68	MMP8:1346:21	TGTTCTTGCTGGAAAACTGCA	21	43	56.2
69	MMP9:1677:21	ACTTGTCGGCGATAAGGAAGG	21	52	57.4
70	MMP9:2062:19	CTCACTCCGGGAACTCACG	19	63	57.2
71	PLAU:444:19	CTCCGGTTGTCTGGGTTCC	19	63	57.3
72	PLAU:1366:18	CCTGGGGACCCTCAGAGG	18	72	57.6
73	PLAUR:1008:20	CCTCGTTGCATTTGGTGGTG	20	55	57.4
74	PLAUR:1365:21	GTGATGGTGAGGCTGAGATGG	21	57	57.8
75	SERPINB2:952:22	CCATTTTGTCTTTGCTGGTCCA	22	45	56.9
76	SERPINB2:1268:24	AAATGCAGTTGGTTATCTTATGCA	24	33	54.7
77	SERPINE1:1106:21	CCTCGATCTTCACTTTCTGCA	21	48	55.4
78	SERPINE1:1291:20	CCAGATGAAGGCGTCTTTCC	20	55	56.0
79	spikeB:3628:18	CTGCAGCTGCGTGTTCTG	18	61	56.9
80	spikeB:4259:21	GTTTTGCGCACTCTTTCTCGT	21	48	57.4
81	spikeH:11652:19	GCTGCTCAGCCTTCTTTCG	19	58	56.6
82	spikeH:11875:21	TCAGCGTCGCTTCATAATCCT	21	48	56.9
83	spikeJ:16986:18	CTGTTCACCGCCAGCACA	18	61	58.0
84	spikeJ:17766:23	TGGTTTCAACCTGTCTGATATCC	23	43	55.4
85	ST14:1468:23	GGAGAGGTATTCAGCTAAGAAGC	23	48	55.7
86	ST14:2734:20	CCAAATGTGTACACCTGCGG	20	55	56.8
87	TIMP1:564:18	CTGGTCCGTCCACAAGCA	18	61	56.9
88	TIMP1:570:18	GAGGAGCTGGTCCGTCCA	18	67	58.0
89	TIMP2:661:20	GAGGGTGATGTGCATCTTGC	20	55	56.6
90	TIMP2:877:21	CGTCACTTCTCTTGATGCAGG	21	52	56.4
91	TIMP3:1509:24	GTGTACATCTTGCCATCATAGACG	24	46	56.9
92	TIMP3:1764:19	CCATCCTCGGTACCAGCTG	19	63	56.9
93	TIMP4:411:20	CCATCACTGAGGACCTGACC	20	60	56.8
94	TIMP4:703:22	ACGATGTCAACAAACTCCTTCC	22	45	56.2
min			18	33	54.7
max			24	72	58.0
mean			20.0	52.0	56.7
