# Supplementary material for: An alternative method to amplify RNA without loss of signal conservation for expression analysis with a proteinase DNA microarray in the ArrayTube® format
Source: BMC Genomics. 2006 Jun 12;7:144. doi: 10.1186/1471-2164-7-144 (PMC1526438; doi:10.1186/1471-2164-7-144)
Supplement: Additional file 4 — Probes with reference sequences [file 1471-2164-7-144-S4.pdf]

Additional file 4 – table 4

Probes with reference sequences

i	name	location	distance_3'end	sequence	len	GC
1	ACTB:874:49	NM_001101.2[874:922]	279	GGCATGGAGTCCTGTGGCATCCACGAAACTACCTTCAACTCCATCATGA	49	51
2	ACTB:1120:48	NM_001101.2[1120:1167]	34	TCCACCTTCCAGCAGATGTGGATCAGCAAGCAGGAGTATGACGAGTCC	48	54
3	F3:386:50	NM_001993.2[386:435]	576	GTGTGACCTCACCGACGAGATTGTGAAGGATGTGAAGCAGACGTACTTGG	50	52
4	F3:945:47	NM_001993.2[945:991]	20	CACAAGTGTAGAAAGGCAGGAGTGGGGCAGAGCTGGAAGGAGAACTC	47	55
5	GAPD:829:48	NM_002046.2[829:876]	207	CTGCCAAATATGATGACATCAAGAAGGTGGTGAAGCAGGCGTCGGAGG	48	52
6	GAPD:1010:50	NM_002046.2[1010:1059]	24	CTGGTATGACAACGAATTTGGCTACAGCAACAGGGTGGTGGACCTCATGG	50	52
7	CTSB:211:50	NM_001908.2[211:260]	911	CCCTCTTTCCATCCCCTGTCGGATGAGCTGGTCAACTATGTCAACAAACG	50	52
8	CTSB:1089:51	NM_001908.2[1089:1139]	32	CAGAGGACAGGATCACTGTGGAATCGAATCAGAAGTGGTGGCTGGAATTCC	51	51
9	CTSC:1059:50	NM_001814.2[1059:1108]	382	GCTTCCCCTACACAGGCACTGATTCTCCATGCAAAATGAAGGAAGACTGC	50	50
10	CTSC:1400:50	NM_001814.2[1400:1449]	41	GAGAATGGCTACTTCCGGATCCGCAGAGGAACTGATGAGTGTGCAATTGA	50	50
11	CTSE:356:50	NM_001910.2[356:405]	904	GCACTATCTCCATTGGCTCCCCACCACAGAACTTCACTGTCATCTTCGAC	50	52
12	CTSE:1245:49	NM_001910.2[1245:1293]	16	ACAGTTTTACTCAGTCTTTGACCGTGGGAATAACCGTGTGGGACTGGCC	49	51
13	CTSD:887:50	NM_001909.3[887:936]	337	CCAAGTATTACAAGGGTTCTCTGTCCTACCTGAATGTCACCCGCAAGGCC	50	52
14	CTSD:1163:46	NM_001909.3[1163:1208]	65	GCAAAGGCTACAAGCTGTCCCCAGAGGACTACACGCTCAAGGTGTC	46	57
15	CTSF:767:50	NM_003793.2[767:816]	729	TGGCACAGCTCAGTATGGAGTCACCAAGTTCAGTGATCTCACAGAGGAGG	50	52
16	CTSF:1435:50	NM_003793.2[1435:1484]	61	TCAAGAACAGCTGGGGCACTGACTGGGGTGAGAAGGGTTACTACTACTTG	50	52
17	CTSG:365:50	NM_001911.2[365:414]	391	TGTTATTGCAGCTGAGCAGAAGAGTCAGACGGAATCGAAACGTGAACCCA	50	48
18	CTSG:705:50	NM_001911.2[705:754]	51	TCCTCCAGAAGTCTTCACCAGGGTCTCAAGTTTCCTGCCCTGGATAAGGA	50	52
19	CTSH:695:50	NM_004390.2[695:744]	359	GGTGAAGACACCTACCCCTACCAGGGCAAGGATGGTTATTGCAAGTTCCA	50	52
20	CTSH:1015:48	NM_004390.2[1015:1062]	41	GGGAATGAACGGGTACTTCCTCATCGAGCGCGGAAAGAACATGTGTGG	48	54
21	CTSK:853:50	NM_000396.2[853:902]	212	CCTGTCTCTGTGGCCATTGATGCAAGCCTGACCTCCTTCCAGTTTTACAG	50	52
22	CTSK:947:50	NM_000396.2[947:996]	118	ACCATGCGGTTTTGGCAGTGGGATATGGAATCCAGAAGGGAAACAAGCAC	50	50
23	CTSL:701:50	NM_001912.2[701:750]	596	TGGAGAGAGAAAGGCTACGTGACTCCTGTGAAGAATCAGGGTCAGTGTGG	50	52
24	CTSL:1270:50	NM_001912.2[1270:1319]	27	TGGCTACGTAAAGATGGCCAAAGACCGGAGAAACCATTGTGGAATTGCCT	50	48
25	CTSS:520:50	NM_004079.3[520:569]	560	GTGAAATATCAAGGTTCTTGTGGTGCTTGCTGGGCTTTCAGTGCTGTGGG	50	50
26	CTSS:999:49	NM_004079.3[999:1047]	82	TGGGAAAGAATACTGGCTTGTGAAAAACAGCTGGGGCCACAACTTTGGT	49	47
27	CTSZ:760:50	NM_001336.2[760:809]	228	CAGCTGTGGAATAATGGCAACAGAAAGACTGGCTAACTACACCGGAGGCA	50	50
28	CTSZ:981:46	NM_001336.2[981:1026]	11	CCAGATACAACCTTGCCATCGAGGAGCACTGTACATTTGGGGACCC	46	54

29	ST14:1416:50	NM_021978.2[1416:1465]	1255	GCAACAAGATCACAGTTCGCTTCCACTCAGATCAGTCCTACACCGACACC	50	52
30	ST14:2664:49	NM_021978.2[2664:2712]	8	TGTACACAAGGCTCCCTCTGTTTCGGGACTGGATCAAAGAGAACACTGG	49	51
31	MMP1:1120:50	NM_002421.2[1120:1169]	312	TGTTCAGGGACAGAATGTGCTACACGGATACCCCAAGGACATCTACAGCT	50	50
32	MMP1:1290:54	NM_002421.2[1290:1343]	138	TGGATCCAGGTTATCCCAAAATGATAGCACATGACTTTCCTGGAATTGGCCACA	54	44
33	MMP2:1983:50	NM_004530.1[1983:2032]	240	CCAGCGAGTGGATGCCGCCTTTAACTGGAGCAAAAACAAGAAGACATACA	50	48
34	MMP2:2219:50	NM_004530.1[2219:2268]	4	GTCTGAAGAGCGTGAAGTTTGGAAGCATCAAATCCGACTGGCTAGGCTGC	50	52
35	MMP3:725:50	NM_002422.2[725:774]	723	CCACTCCCTGGGTCTCTTTCACTCAGCCAACACTGAAGCTTTGATGTACC	50	52
36	MMP3:1316:49	NM_002422.2[1316:1364]	133	GGAGCCAGGCTTTCCCAAGCAAATAGCTGAAGACTTTCCAGGGATTGAC	49	51
37	MMP7:275:50	NM_002423.2[275:324]	527	CGCGTCATAGAAATAATGCAGAAGCCCAGATGTGGAGTGCCAGATGTTGC	50	50
38	MMP7:676:50	NM_002423.2[676:725]	126	TGCTGCAACTCATGAACTTGGCCATTCTTTGGGTATGGGACATTCCTCTG	50	48
39	MMP8:753:50	NM_002424.1[753:802]	673	CCTCTGACCCTGGTGCCTTGATGTATCCCAACTATGCTTTCAGGGAAACC	50	52
40	MMP8:1276:51	NM_002424.1[1276:1326]	149	ACAATTCATGGAGCCAGGTTATCCCAAAAGCATATCAGGTGCCTTTCCAGG	51	47
41	MMP9:1592:50	NM_004994.1[1592:1641]	502	CGGAGATTGGGAACCAGCTGTATTTGTTCAAGGATGGGAAGTACTGGCGA	50	50
42	MMP9:2008:51	NM_004994.1[2008:2058]	85	GTCTTCCAGTACCGAGAGAAAGCCTATTTCTGCCAGGACCGCTTCTACTGG	51	53
43	MMP10:718:50	NM_002425.1[718:767]	686	GCTTTGATGTACCCACTCTACAACTCATTCACAGAGCTCGCCCAGTTCCG	50	52
44	MMP10:1371:50	NM_002425.1[1371:1420]	33	TGGATCATCACAGTTTGAGTTTGACCCCAATGCCAGGATGGTGACACACA	50	48
45	MMP11:1214:47	NM_005940.2[1214:1260]	229	ACAAGATCTACTTCTTCCGAGGCAGGGACTACTGGCGTTTCCACCCC	47	55
46	MMP11:1380:47	NM_005940.2[1380:1426]	63	CCTCTACTGGAAGTTTGACCCTGTGAAGGTGAAGGCTCTGGAAGGCT	47	53
47	MMP12:845:50	NM_002426.1[845:894]	531	ACCAGCTCTCTGTGACCCCAATTTGAGTTTTGATGCTGTCACTACCGTGG	50	50
48	MMP12:1243:50	NM_002426.1[1243:1292]	133	ACCCTGGTTATCCCAAACTGATTACCAAGAACTTCCAAGGAATCGGGCCT	50	48
49	MMP13:766:50	NM_002427.2[766:815]	629	ACCGGCAAAAGCCACTTTATGCTTCCTGATGACGATGTACAAGGGATCCA	50	48
50	MMP13:1390:51	NM_002427.2[1390:1440]	4	TGGAGTAACCGTATTGTTCGCGTCATGCCAGCAAATTCCATTTTGTGGTGT	51	45
51	MMP14:558:50	NM_004995.2[558:607]	1376	AGGAAGCGCTACGCCATCCAGGGTCTCAAATGGCAACATAATGAAATCAC	50	48
52	MMP14:1784:47	NM_004995.2[1784:1830]	153	GACTGAGGAGGAGACGGAGGTGATCATCATTGAGGTGGACGAGGAGG	47	57
53	MMP15:451:51	NM_002428.1[451:501]	1557	CCGGGAGGAAGTGGAACAACCACCATCTGACCTTTAGCATCCAGAACTACA	51	51
54	MMP15:2011:40	NM_002428.1[2011:2050]	8	CACGTGTCCTGCTTTACTGCAAGCGCTCGCTGCAGGAGTG	40	60
55	MMP16:367:50	NM_005941.2[367:416]	1520	GGAAAAGTGGACAGAAACACAATTGACTGGATGAAGAAGCCCCGATGCGG	50	50
56	MMP16:1866:50	NM_005941.2[1866:1915]	21	CCAGTTCAAGAGGAAAGGAACACCCCGCCACATACTGTACTGTAAACGCT	50	50
57	MMP17:657:46	NM_016155.2[657:702]	1218	GACATCCAGATCGACTTCTCCAAGGCCGACCATAACGACGGCTACC	46	57
58	MMP17:1573:46	NM_016155.2[1573:1618]	302	CCTCCTACTTCTTCCGTGGCCAGGAGTACTGGAAAGTGCTGGATGG	46	57
59	MMP19:1254	NM_002429.2[1	332	GCTCTCTATTGGCCTCTCAACCAAAAGGT	50	52

	:50	254:1303]		GTTCCTCTTTAAGGGCTCCGG		
60	MMP19:1570:50	NM_002429.2[1570:1619]	16	CCTCAGGTACGGGCATAACCTTGGATACCACTCTCTCAGCCACAGAAACC	50	54
61	MMP24:1148:50	NM_006690.2[1148:1197]	744	CAACTTCAACACAGTGGCCCTCTTCCGGGGCGAGATGTTTGTCTTTAAGG	50	52
62	MMP24:1852:48	NM_006690.2[1852:1899]	42	TGCTGGTCTACACCATCTTCCAGTTCAAGAACAAGACAGGCCCTCAGC	48	52
63	TIMP1:370:50	NM_003254.1[370:419]	267	CGAGGAGTTTCTCATTGCTGGAAAACTGCAGGATGGACTCTTGCACATCA	50	48
64	TIMP1:505:50	NM_003254.1[505:554]	132	GGAATGCACAGTGTTTCCCTGTTTATCCATCCCCTGCAAACTGCAGAGTG	50	50
65	TIMP2:604:50	NM_003255.2[604:653]	312	GCTGGACGTTGGAGGAAAGAAGGAATATCTCATTGCAGGAAAGGCCGAGG	50	52
66	TIMP2:809:47	NM_003255.2[809:855]	110	GACGAGTGCCTCTGGATGGACTGGGTCACAGAGAAGAACATCAACGG	47	55
67	TIMP3:1448:50	NM_000362.3[1448:1497]	327	CGAGAGTCTCTGTGGCCTTAAGCTGGAGGTCAACAAGTACCAGTACCTGC	50	54
68	TIMP3:1685:50	NM_000362.3[1685:1734]	90	CGACATGCTCTCCAATTTCGGTTACCCTGGCTACCAGTCCAAACACTACG	50	52
69	TIMP4:342:50	NM_003256.1[342:391]	343	CGCCTTTTGACTCTTCCCTCTGTGGTGTGAAACTAGAAGCCAACAGCCAG	50	52
70	TIMP4:622:50	NM_003256.1[622:671]	63	TGGTTACCAGGCTCAGCATTATGTCTGTATGAAGCATGTTGACGGCACCT	50	48
71	PLAU:392:50	NM_002658.1[392:441]	931	CCCACAGATCTGATGCTCTTCAGCTGGGCCTGGGGAAACATAATTACTGC	50	52
72	PLAU:1309:50	NM_002658.1[1309:1358]	14	GTCTCACACTTCTTACCCTGGATCCGCAGTCACACCAAGGAAGAGAATGG	50	52
73	PLAUR:954:50	NM_002659.1[954:1003]	431	GGCTCCAATGGTTTCCACAACAACGACACCTTCCACTTCCTGAAATGCTG	50	50
74	PLAUR:1284:50	NM_002659.1[1284:1333]	101	TGCTGTACTAAAAGTGGCTGTAACCACCCAGACCTGGATGTCCAGTACCG	50	52
75	SERPINE1:1042:50	NM_000602.1[1042:1091]	193	GACAGTTTCAGGCTGACTTCACGAGTCTTTCAGACCAAGAGCCTCTCCAC	50	52
76	SERPINE1:1233:47	NM_000602.1[1233:1279]	5	CACAACCCCACAGGAACAGTCCTTTTCATGGGCCAAGTGATGGAACC	47	53
77	SERPINB2:888:50	NM_002575.1[888:937]	383	GCCGATGTGTCCACTGGCTTGGAGCTGCTGGAAAGTGAAATAACCTATGA	50	50
78	SERPINB2:1189:50	NM_002575.1[1189:1238]	82	CAGGAGGTGTTATGACAGGGAGAACTGGACATGGAGGCCCACAGTTTGTG	50	54
79	CSTA:86:50	NM_005213.2[86:135]	219	ACCCGCCACTCCAGAAATCCAGGAGATTGTTGATAAGGTTAAACCACAGC	50	48
80	CSTA:271:51	NM_005213.2[271:321]	33	GTCTTCCCGGACAAAATGAGGACTTGGTACTTACTGGATACCAGGTTGACA	51	47
81	CSTB:203:50	NM_000100.2[203:252]	154	ACAAGAAGTTCCCTGTGTTTAAGGCCGTGTCATTCAAGAGCCAGGTGGTC	50	50
82	CSTB:303:50	NM_000100.2[303:352]	54	ACACCTGCGAGTGTTCCAATCTCTCCCTCATGAAAACAAGCCCTTGACCT	50	50
83	CSTC:310:47	NM_000099.2[310:356]	160	GCAAGCAGATCGTAGCTGGGGTGAACTACTTCTTGGACGTGGAGCTG	47	55
84	CSTC:393:50	NM_000099.2[393:442]	74	GACAACTGCCCCTTCCATGACCAGCCACATCTGAAAAGGAAAGCATTCTG	50	50
85	CST5:173:50	NM_001900.2[173:222]	211	AGGTCATTAATAAGGATGAGTACTACAGCCGCCCTCTGCAGGTGATGGCT	50	50
86	CST5:300:50	NM_001900.2[300:349]	84	GCCCAACTTGGACAACTGTCCCTTCAATGACCAGCCAAAACTGAAAGAGG	50	50
87	KNG:978:50	NM_000893.1[978:1027]	306	GGTGGTGGCTGGCAAGAAATATTTTATTGACTTCGTGGCCAGGGAAACCA	50	48
88	KNG:1150:50	NM_000893.1[1150:1199]	134	GTCAACTGTCAACCACTGGGAATGATCTCACTGATGAAAAGGCCTCCAGG	50	50
89	spikeB:399:50	spikeB[399:448]		CGGTGCAAATGTGTTTTACAGCGTGATGGAGCAGATGAAGATGCTCGACA	50	48

90	spikeB:1025:50	spikeB[1025:1074]	CGATGGTCTGAAAGAAGTTCAGGAAGCGGTGATGCTGATAGAAGCCGGAC	50	52
91	spikeH:65:50	spikeH[65:114]	TGATTATTTTGTTAACGATGAAGCGCGGGCGCGTTACTGGGATGATCGTG	50	48
92	spikeH:297:50	spikeH[297:346]	CTGAACAAGGCACTGAAAGACGGGAAAATCCTGCAGGCGGATTACAACAC	50	50
93	spikeJ:634:50	spikeJ[634:683]	ACCGGGCGATGTTATTGAAATCTGCGATGATGACTATGCCGGTATCAGCA	50	48
94	spikeJ:1416:50	spikeJ[1416:1465]	TGCCGTTTATGACCCGACGGTACAGTTTGAGTTCTGGTTCTCGGAAAAGC	50	50
